# Tumor Suppressor LncRNA on Chromosome 8p12 (TSLNC8): A Concise Review in Human Malignancies

**DOI:** 10.7150/jca.87801

**Published:** 2023-09-11

**Authors:** Xia Li, He Huang, Meichen Liu, Hongliang Luo

**Affiliations:** 1 Department of Gastrointestinal Surgery, The Second Affiliated Hospital of Nanchang University, Nanchang 330008, Jiangxi, China.; 2 Department of Spleen and Stomach Diseases, Jiujiang Hospital of Traditional Chinese Medicine, Jiujiang 332005, Jiangxi, China.; 3 Second School of Clinical Medicine, Nanchang University, Nanchang 330038, Jiangxi, China.

**Keywords:** LncRNA, TSLNC8, Malignancy, Tumor biomarker, Therapeutic target

## Abstract

Tumor Suppressor Long Non-Coding RNA on Chromosome 8p12 (TSLNC8) is an RNA gene that generates a long non-coding RNA transcribed intergenically from both strands. Its significant role in human malignancies attracted significant attention in recent years. Expression analysis of TSLNC8 has been conducted in tissue specimens and cell lines using various techniques, including reverse transcription-quantitative polymerase chain reaction (RT-qPCR), *in situ* hybridization (ISH), and microarray analysis. Furthermore, functional studies involving the loss and/or gain of TSLNC8 function in cellular and animal models have been carried out. These investigations have highlighted the impact of TSLNC8 on key tumor-related processes, including migration, invasion, and metastasis. Moreover, TSLNC8 has emerged as a regulator capable of modulating critical signaling pathways, such as the Hippo, STAT3, WNT/β-catenin, and MAPK pathways. In this review, we comprehensively synthesize the findings derived from *in vitro* and *in vivo* studies, along with analyses conducted on clinical samples, to provide a comprehensive understanding of the multifaceted role of TSLNC8 as a promising tumor biomarker and a potential target for therapeutic interventions.

## Introduction

Noncoding RNAs (ncRNAs) constitute a substantial portion (~98%) of the human transcriptome and are functionally divided into two distinct types, housekeeping and regulatory ncRNAs 1-3. Long noncoding RNAs (lncRNAs) represent a key subclass of regulatory ncRNAs that exceed 200 nucleotides in length and lack substantial protein-coding capacity 4, 5. Emerging as shining star, lncRNAs actively participate in chromatin remodeling, transcriptional, and posttranscriptional events 6, 7. Through interactions with DNA, RNA, and protein molecules, lncRNAs contribute to intricate regulatory networks that are involved in a wide range of cellular processes and pathological functions 8-11. Recent evidence continues to accumulate, shedding light on the pivotal roles of lncRNAs as either oncogenes or tumor suppressors, underscoring their potential as therapeutic targets in various disorders, with a particular emphasis on cancer 12-18.

**Figure 1:**
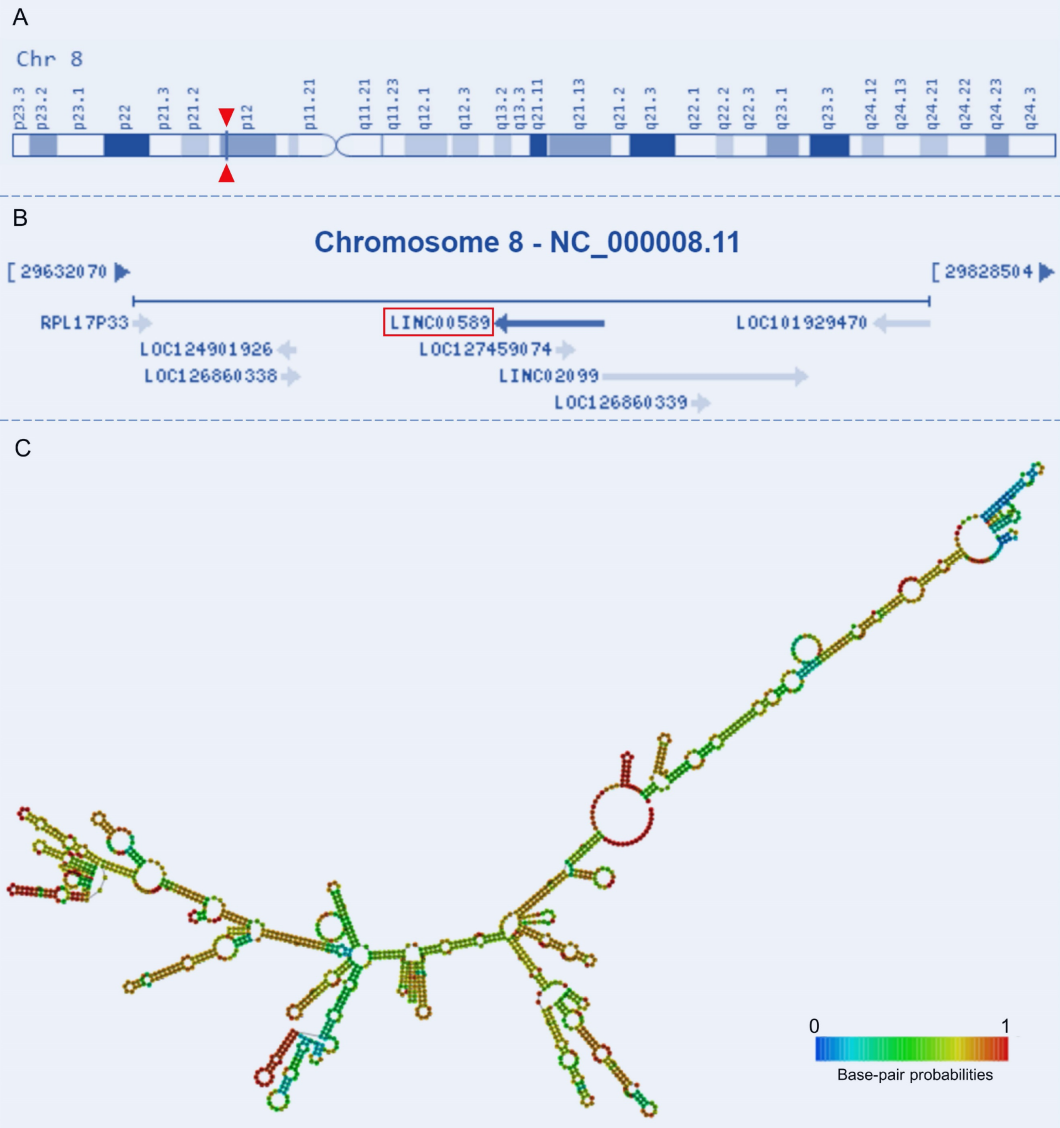
Genomic view and structure of LINC00589: **(A)** genomic location extracted from GeneCards database (https://www.genecards.org/cgi-bin/carddisp.pl?gene=LINC00589), **(B)** genomic context from NCBI database (https://www.ncbi.nlm.nih.gov/gene/619351), **(C)** minimum free energy secondary structure extracted from RNAfold web server (http://rna.tbi.univie.ac.at//cgi-bin/RNAWebSuite/RNAfold.cgi?PAGE=3&ID=PEEiuT7Ten), colored by base-pairing probability.

Tumor Suppressor LncRNA on Chromosome 8p12 (TSLNC8), also known as Long Intergenic Non-Protein Coding RNA 589 (LINC00589) or Chromosome 8 Open Reading Frame 75 (C8orf75), is located on Chromosome 8p12 and comprises four exons that are non-overlapping with annotated coding genes (**Figure 1A-B**). With a total length of 1413 bp, its secondary structure has also been characterized (**Figure 1C**). This lncRNA has been validated as a non-protein-coding RNA and has emerged as a key player in tumorigenesis^19^, ^20^. Altered expression of TSLNC8 has been detected in multiple tumorous tissues and cancer cell lines 19-22. It also participates in a wide range of tumor-related processes and regulates drug resistance and the progression of a variety of human malignancies 19-28. Given the observed dysregulation of this lncRNA TSLNC8 in diverse malignancies, and its reported significant associations with clinicopathology and survival outcomes 19, 20, 23, 24, 26, 27, TSLNC8 presents a promising prospect as a potential therapeutic target. Therefore, it is imperative to ascertain the molecules and pathways associated with this lncRNA in different tumor contexts. This manuscript provides a comprehensive summary of pertinent studies, categorized into three sections: investigations conducted on cell lines, studies utilizing animal models, and analyses performed on clinical samples.

### TSLNC8 in cancers

**Figure 2:**
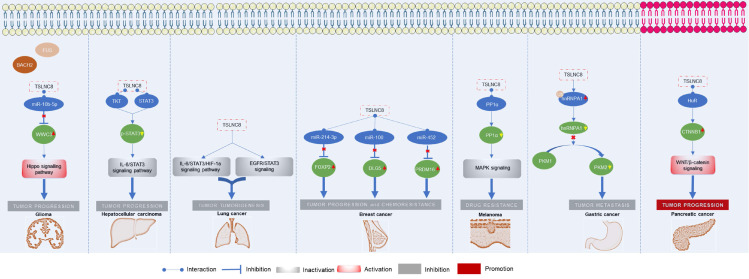
Functional mechanism of TSLNC8 as a tumor suppressor or oncogene in the initiation and progression of different tumors.

**Figure 3:**
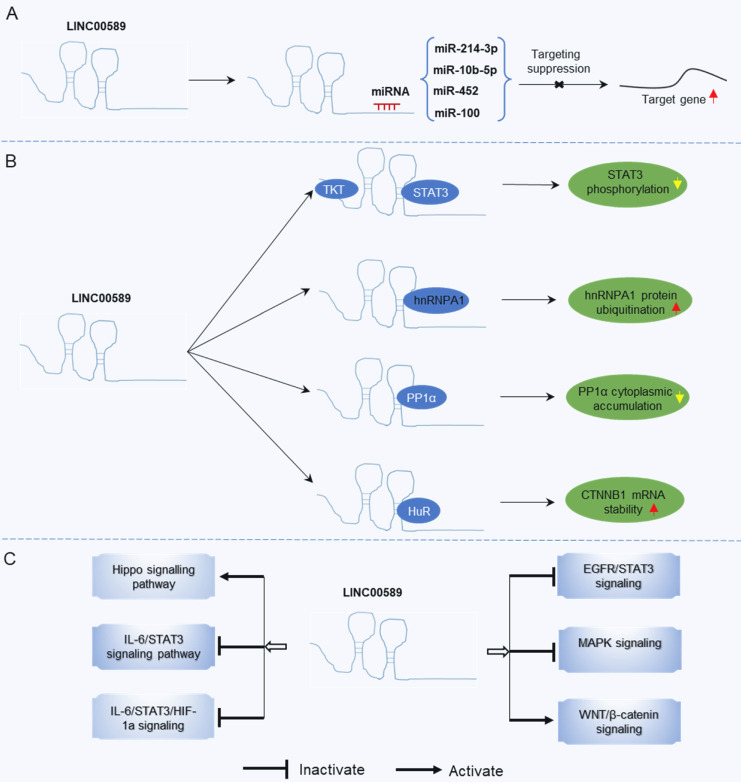
Mechanisms underlying the regulatory role of LINC00589 in tumor progression. **(A)** LINC00589 functions as a ceRNA, **(B)** LINC00589 engages in physical interactions with proteins, **(C)** LINC00589 participates in the modulation of signaling pathways.

The roles of TSLNC8 have been investigated across multiple cancer types, as depicted in **Figure 2**. It exhibits diverse functions depending on the specific cancer context. In glioma 20, liver cancer 19, lung cancer 27, breast cancer 21, melanoma 25, and gastric cancer 23, TSLNC8 consistently acts as a tumor suppressor, effectively inhibiting tumor progression and reducing chemoresistance when overexpressed. However, in pancreatic cancer 26, the dysregulated expression of LINC00589 functions as an oncogenic driver, promoting tumor progression and metastasis. Overall, TSLNC8 functions as a regulatory molecule in tumor progression by acting as a competitive endogenous RNA (ceRNA), sequestering specific microRNAs (**Figure 3A**). Furthermore, it engages in interactions with specific proteins, regulation of target protein phosphorylation, ubiquitination, cytoplasmic accumulation, or stability of target mRNA (**Figure 3B**). Moreover, TSLNC8 is implicated in multiple signaling pathways, as depicted in **Figure 3C**. The functional implications of TSLNC8 *in vitro* and/or *in vivo* experiments are discussed in detail below.

### *In vitro* cell line assays

**Figure 4:**
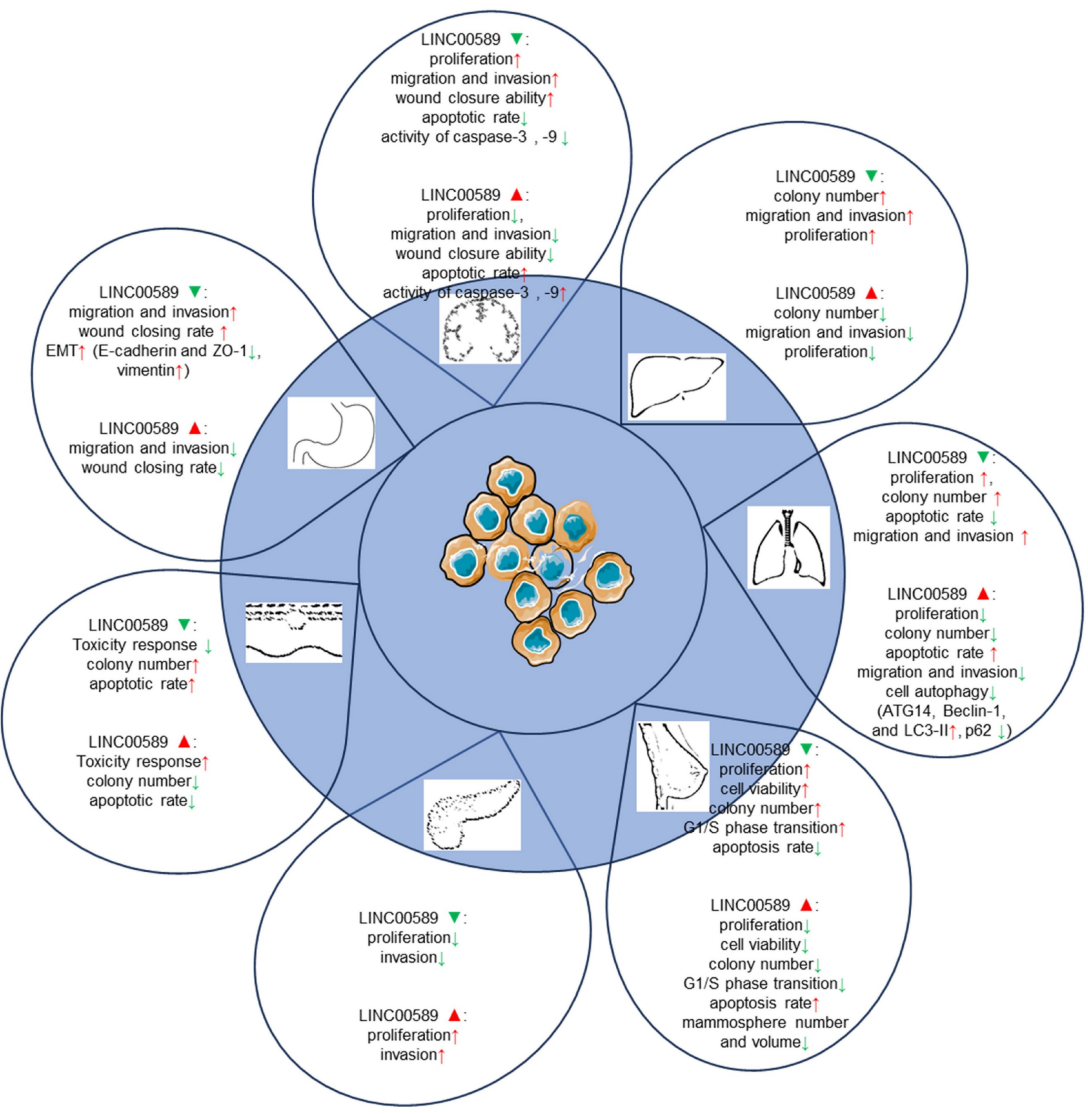
Functions of TSLNC8 upregulation and silencing in cell-based assays.

The *in vitro* studies conducted on TSLNC8 across different cancer types have provided valuable insights into its functional effects. These findings are summarized in** Table 1** and illustrated in **Figure 4**.

In glioma cells, TSLNC8 overexpression resulted in decreased cell proliferation, inhibition of migration and invasion, and an increase in apoptotic rate, while TSLNC8 knockdown exhibited the opposite effects 20, 28. The suppressive effects of TSLNC8 overexpression are mediated through competitive endogenous RNA (ceRNA) interactions with miR-10b-5p, which attenuates the repression of WWC3 by miR-10b-5p and activates the Hippo signaling pathway 28.

*In vitro* experiments in HCC cancer cells have revealed that overexpression of

TSLNC8 via lentiviral infection resulted in a marked suppression of colony formation and reduced proliferation rates. Conversely, silencing TSLNC8 accelerated colony formation and increased cell proliferation. TSLNC8 overexpression effectively inhibited the migration and invasion of HCC cells, while its knockdown enhanced these cellular processes. Mechanistically, TSLNC8 physically interacts with TKT and STAT3, leading to the inhibition of STAT3 phosphorylation and transcriptional activity. This interaction ultimately results in the inactivation of the IL-6/STAT3 signaling pathway, thereby contributing to the tumor-suppressive effects of TSLNC8 in HCC.

In lung cancer cells 22, 27, TSLNC8 displayed significant downregulation, whereas its overexpression resulted in the suppression of autophagy and exerted inhibitory effects on cell migration, invasion, and apoptosis promotion. Conversely, TSLNC8 knockdown showed opposite effects. Moreover, TSLNC8 exhibited a remarkable ability to inhibit the aggressive behaviors of lung cancer cells by targeting the IL-6/STAT3/HIF-1a signaling pathway 22. Additionally, a synergistic effect was observed between TSLNC8 and the EGFR inhibitor osimertinib, effectively suppressing lung cancer tumorigenesis by blocking the EGFR-STAT3 pathway 27.

In breast cancer 21, up-regulation of TSLNC8 has been shown to decrease the proliferation capacity of breast cancer cells and inhibit the transition from G1 to S phase of the cell cycle. Conversely, TSLNC8 knockdown exhibited the opposite effect. These effects are mediated through the miR-214-3p/FOXP2 axis. In HER2+ breast cancer 24, LINC00589 played a crucial role in enhancing the sensitivity of breast cancer cells to trastuzumab and suppressing anchorage-independent growth. The expression of LINC00589 also reversed cancer stem cell-like properties and reduced chemoresistance in HER2-positive breast cancer. Acting as a ceRNA platform, LINC00589 acts as a sponge for miR-100 and miR-452, thus relieving their suppression of tumor suppressors such as DLG5 and PRDM16. Through this mechanism, LINC00589 exerted multiple inhibitory functions on cancer progression and effectively counteracts drug resistance.

In pancreatic cancer cell lines 26, knockdown of TSLNC8 suppressed cell proliferation and attenuated invasiveness, while overexpression of TSLNC8 increased cell proliferation and enhanced invasion. TSLNC8 interacted with HuR, facilitating HuR's binding to CTNNB1 mRNA and enhancing its stability, ultimately activating the WNT/β-catenin signaling pathway and promoting aggressiveness in pancreatic cancer cells.

In melanoma 25, TSLNC8 overexpression sensitized cells to the BRAF inhibitor PLX4720, promoting apoptosis and reducing colony formation. TSLNC8 downregulation had the opposite effect, decreasing sensitivity to the inhibitor and increasing colony formation. TSLNC8 achieved its pro-sensitivity effect by binding to PP1α, leading to decreased cytoplasmic accumulation and modulation of MAPK signaling.

In gastric cancer 23, LINC00589 exhibited suppressive effects on migration and invasion of GC cells *in vitro*. Silencing LINC00589 enhanced the invasion and migration abilities of cancer cells and induced epithelial-mesenchymal transition (EMT). LINC00589 interacted with hnRNPA1 protein, leading to its ubiquitination and degradation, consequently inhibiting PKM2 isoform generation and suppressing carcinogenesis.

### *In vivo* mouse model experiments

**Figure 5:**
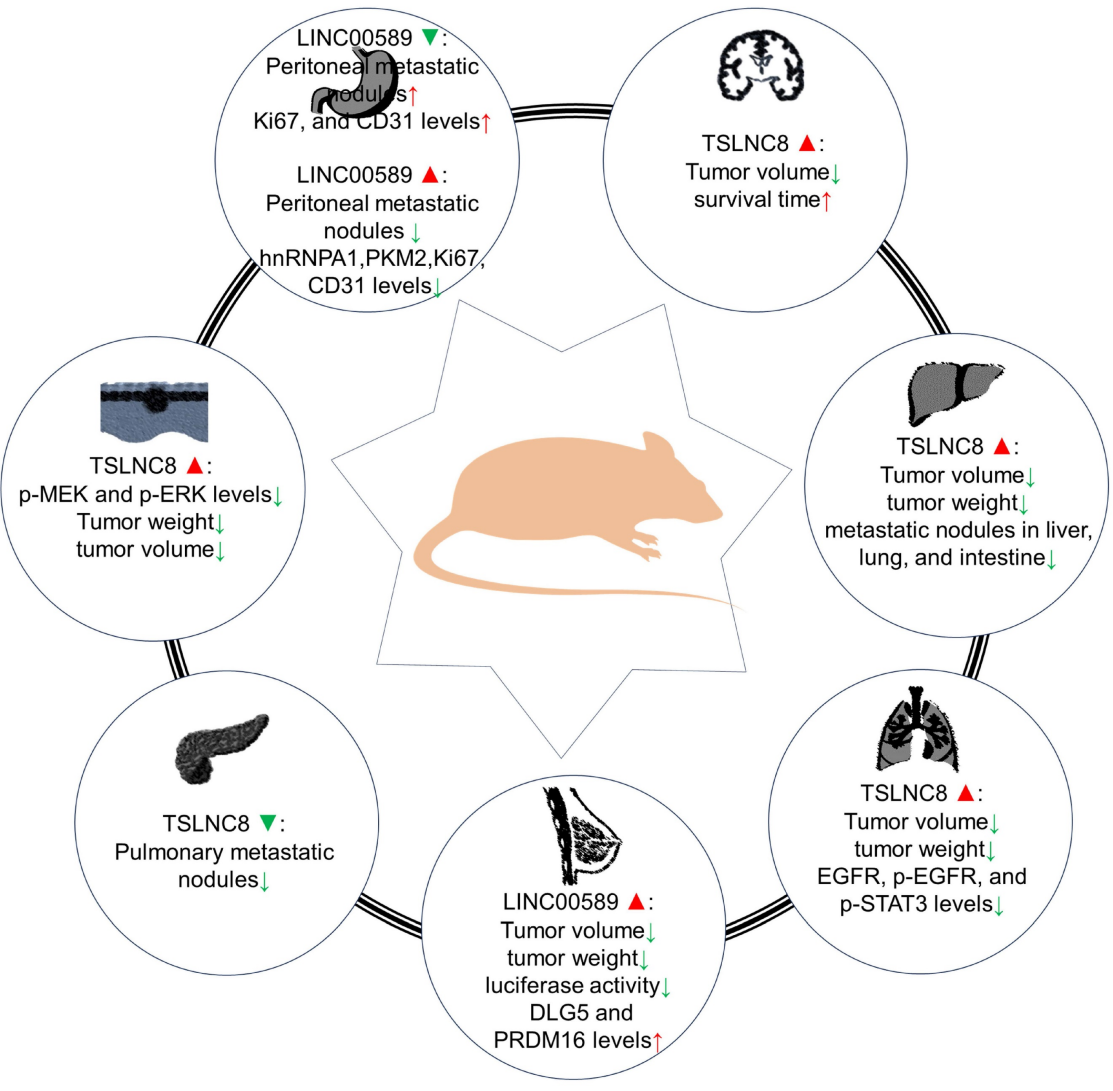
Roles of TSLNC8 overexpression and/or knockdown in tumorigenesis in mouse xenograft models.

Multiple research teams have investigated the functional implications of LINC00589 up-regulation and/or silencing on tumor development using xenograft models (**Table 2** and **Figure 5**). Similar to *in vitro* studies, both oncogenic and tumor suppressor role have been reported for LINC00589.

Animal models have provided evidence regarding the impact of LINC00589 modulation on different cancer types. In pancreatic cancer, LINC00589 knockdown reduced pulmonary metastatic nodules 26. Whereas in glioma models 28, it was demonstrated that knockdown of BACH2 or FUS, overexpression of TSLNC8, or a combination of the three inhibited subcutaneous xenograft growth and prolonged survival in nude mice. In HCC 19, its upregulation was associated with decreased tumor volume, weight, and fewer metastatic nodules in the liver, lung, and intestine. In lung cancer 27, TSLNC8 overexpression led to smaller tumor volume and weight, reduced expression of EGFR, p-EGFR, and p-STAT3 levels, and combination with osimertinib administration effectively suppressed tumor growth, enhancing osimertinib's anti-tumor effects. In HER2+ breast cancer 24, LINC00589 overexpression significantly decreased tumor volume and weight, inhibited luciferase activity, and upregulated DLG5 and PRDM16 protein expressions. Additionally, LINC00589 reversed trastuzumab resistance through miR-100 and miR-452 in breast cancer, as confirmed in xenograft nude mouse models 24. In melanoma 25, TSLNC8 overexpression diminished tumor growth rate and weight and enhanced the cytotoxic effects of the BRAF inhibitor PLX4720. In gastric cancer 23, LINC00589 knockdown resulted in peritoneal metastatic nodules and decreased Ki67 and CD31 protein levels, while its overexpression reduced the number of peritoneal metastatic nodules and decreased hnRNPA1, PKM2, Ki67, and CD31 levels.

### Ex vivo clinical sample studies

Based on the existing studies, TSLNC8 expression levels exhibit diverse implications in different types of cancer. In pancreatic cancer, Chai et al. 26 reported that upregulation of TSLNC8 in cancerous tissues, which was significantly associated with advanced TNM stage, lymph node and distant metastasis, and poorer overall survival (OS). However, in most studies, TSLNC8 was found to be downregulated in cancer samples. In glioma, downregulation of TSLNC8 was linked to larger tumor size, distant metastasis, and higher TNM stage. In hepatocellular carcinoma (HCC), Zhang et al 19 found that TSLNC8 was frequently deleted and downregulated, and lower levels of TSLNC8 RNA were correlated with an increased number of tumor nodules, presence of cancer embolus, poorer differentiation stage, and shorter OS in tumor cases. In lung cancer, the expression level of TSLNC8 was correlated with gender, lymph node metastasis, and TNM stage. In breast cancer, TSLNC8 was downregulated in cancerous tissues compared to adjacent normal tissues. In HER2+ breast cancer, lower expression of LINC00589 was associated with non-response to trastuzumab, advanced TNM stage, shorter survival time and acted as an independent unfavorable prognostic factor for OS. In gastric cancer, TSLNC8 was inversely associated with aggressive pathological features, tumor prognosis, and was an independent prognostic factor. **Table 3** presents a detailed summary of the expression of TSLNC8 and its associations with pathological features and prognosis in clinical tumor samples.

**Figure 6:**
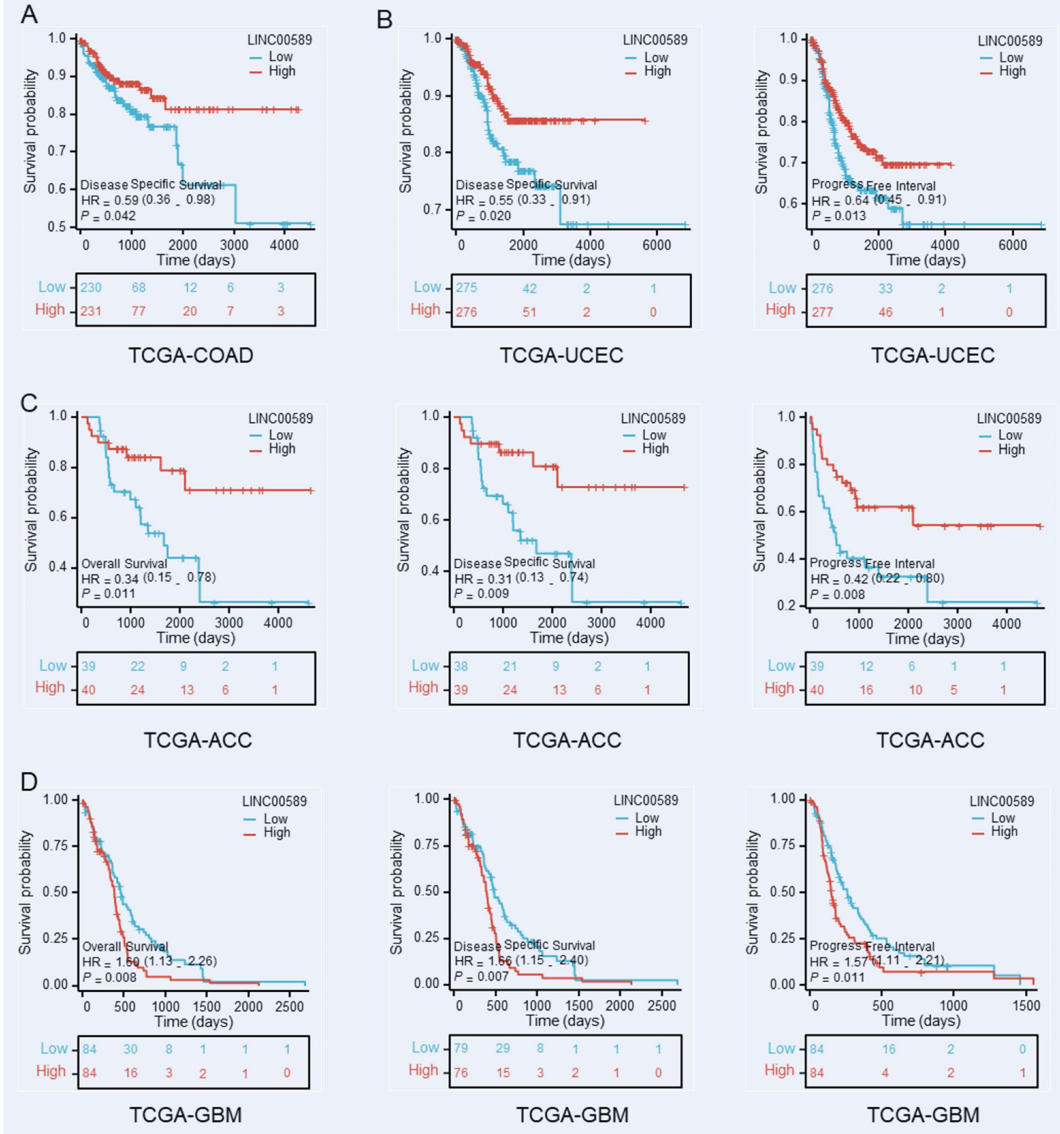
Kaplan-Meier survival curves demonstrate the substantial prognostic significance associated with decreased expression of LINC00589 in COAD **(A)**, UCEC **(B)**, ACC **(C)**, and GBM **(D)**.

Furthermore, we investigated the relationship between LINC00589 and prognosis in various cancer types using data from The Cancer Genome Atlas (TCGA) database (https://portal.gdc.cancer.gov/), focusing on OS, disease-specific survival (DSS), and progression-free interval (PFI). Our analysis revealed significant correlations between LINC00589 expression levels and patient prognosis in colorectal adenocarcinoma (COAD), uterine corpus endometrial carcinoma (UCEC), adrenocortical carcinoma (ACC), and glioblastoma multiforme (GBM) (**Figure 6**). Specifically, low expression of LINC00589 was associated with worse DSS in COAD, shorter DSS and PFI in UCEC, inferior OS, DSS, and PFI in ACC, while better OS, DSS, and PFI in GBM. These findings indicate that the role of LINC00589 may vary depending on the specific cancer type, and its expression levels have the potential to serve as valuable prognostic indicators in certain malignancies.

## Discussion

Deletion of the short arm of chromosome 8 (8p) is a recurrent genetic abnormality observed across diverse range of cancer types 29, 30. It is considered one of the most prevalent genetic events associated with oncogenesis. The loss of genetic material on 8p has been identified in numerous malignancies 31-35, including breast cancer 31, colorectal cancer 36, 37, prostate cancer 38, lung cancer 39, bladder cancer 33, 40, and liver cancer 41. This deletion is implicated in tumor initiation, progression, and clinical outcomes. A recently discovered long non-coding RNA (lncRNA) called TSLNC8, which is located at the 8p12 region, has emerged as a potentially significant contributor in human tumors. This lncRNA has garnered attention for its genomic location within the same chromosomal region where frequent deletions occur in various cancer types.

LINC00589 demonstrates consistent anti-oncogenic properties across various tumor types, except for a single study focusing on pancreatic cancer. However, the precise role of LINC00589 in carcinogenesis remains uncertain, particularly in terms of its tissue-specific functions and whether the findings in pancreatic cancer deviate from its overall tumor-suppressive effects. The primary mechanism underlying the tumor-inhibitory effects of upregulated LINC00589 involves its ability to sequester oncogenic miRNAs. Notably, LINC00589 interacts with multiple miRNAs, such as miR-10b-5p, miR-214-3p, miR-100, and miR-452. Through modulation of these miRNAs, LINC00589 upregulates the expression of tumor suppressor genes such as WWC3, FOXP2, DLG5, and PRDM16. Moreover, LINC00589 interacts physically with proteins involved in the regulation of STAT3 phosphorylation, hnRNPA1 ubiquitination, and PP1α cytoplasmic accumulation. LINC00589 also exerts influence over several signaling pathways, including Hippo, IL-6/STAT3, IL-6/STAT3/HIF-1a, EGFR/STAT3, and MAPK signaling. Additionally, LINC00589 overexpression promotes tumor apoptosis and inhibits metastasis by activating autophagy and suppressing the EMT.

LINC00589 has emerged as a critical mediator of drug resistance in multiple cancer types, including lung cancer 27, melanoma 25, and HER2-positive breast cancer 24. Several researches have demonstrated the role of LINC00589 in regulating chemoresistance and resistance to targeted therapies. Notably, in melanoma 25, the upregulation of LINC00589 has been demonstrated to restore sensitivity to the BRAF inhibitor PLX4720 in resistant cells, offering a potential therapeutic avenue for patients resistant to BRAF inhibitors. Similarly, LINC00589 acts as a modulator, enhancing the efficacy of osimertinib in suppressing the progression of lung cancer 27. Combinatorial treatment involving the overexpression of TSLNC8 and administration of osimertinib has exhibited substantial inhibition of tumor growth in preclinical models 27. Furthermore, LINC00589 has been found to sensitize HER2-positive breast cancer cells to trastuzumab and counteract cancer stem cell-like properties, as well as chemoresistance, to various agents including 5-Fluorouracil, doxorubicin, paclitaxel, cisplatin, gemcitabine, and vincristine^24^. These observations suggest that LINC00589 overexpression could potentially lead to a reduction in tumor volume, attenuation of malignant characteristics, and enhanced responsiveness to chemotherapy and targeted therapies. However, the translation of these promising preclinical findings into clinical practice is impeded by obstacles pertaining to safety and bioavailability. Moreover, LINC00589 exhibits intricate interactions with diverse biomolecules, including miRNAs, and plays regulatory effects on multiple signaling pathways such as STAT3 and MAPK, thereby regulating drug resistance. Identification of additional tissue-specific targets will be critical for the development of more targeted therapeutics.

Interestingly, dysregulated expression of LINC00589 has been associated with patient clinical outcomes, indicating its potential as a clinically significant biomarker. Abnormal expression of LINC00589 has shown significant correlations with tumor development, including tumor grade, lymph node and distant metastasis, and tumor stage. Moreover, aberrant LINC00589 expression has been linked to patient survival and disease progression. Despite the availability of extensive data on the prognostic impact of LINC00589, its diagnostic utility, particularly in invasive body fluids such as serum and urine, remains limited. Future studies should focus on investigating the potential of LINC00589 levels to effectively differentiate between cancer patients and healthy individuals.

In summary, TSLNC8 is a versatile long non-coding RNA that plays a pivotal role in tumor development across multiple cancer types. It exerts regulatory control over crucial tumor-related processes and signaling pathways, and its potential as a tumor biomarker is also underscored, with implications for clinical features and prognostic evaluation. Given its substantial impact on carcinogenesis and treatment response, TSLNC8 emerges as a promising candidate for the development of innovative drugs aimed at improving cancer treatment outcomes. These findings strongly support the potential of TSLNC8 as both a tumor biomarker and a therapeutic target. Further investigation into the underlying mechanisms and therapeutic potential of TSLNC8 holds significant promise for advancing cancer treatment strategies in the future.

## Figures and Tables

**Table 1:** Summary of TSLNC8 expression and roles in tumorigenesis in cancer cell lines.

	Cell expression			*In vitro* experiment cell lines	Cellular functions	Related molecule/ pathway	Ref.
Cancer type	Up or down-regulated	Cell lines	Locations				
Glioma	Downregulated	(U251-MG, SHG-44, BT325, SWO38, CHG-5) vs. a normal astrocyte cell line	-	U251-MG, SWO38, SHG-44, BT325	cell proliferation, migration, invasion, apoptosis	-	20
Glioma	Downregulated	(U87, U251) vs. HA cells	Both the cytoplasm and nucleus	U87, U251	viability, migration, invasion, apoptosis	BACH2, FUS, miR-10b-5p, WWC3, Hippo signaling pathway	28
Hepatocellular carcinoma	Downregulated	Gradually decreases: Huh-7, SNU-449, Hep3B, Huh-6, HepG2, SMMC-7721, SK-Hep1, HCCLM3, PLC/PRC/5, C3A	Mainly distribution in the nucleus	SMMC-7721, SNU-449, Huh-7	cell proliferation, invasion, migration	STAT3, TKT, IL-6, p-STAT3-Y705, P-STAT3-S727, IL-6-STAT3 signaling pathway	19
Non-small cell lung cancer	Downregulated	(A549, H441, H1975) *vs.* HBE	-	A549	cell proliferation, migration, invasion, cell apoptosis, autophagy	Beclin-1, p62, ATG14, and LC3-II, IL-6/STAT3/HIF-1a pathway	22
Lung cancer	Downregulated	(H358, H460, H1975, H1299, H1395, H1650, A549) *vs.* MRC-5	-	H1975, H358	cell proliferation, apoptosis, migration, invasion	EGFR-STAT3 pathway	27
Breast cancer	Downregulated	(MDA-MB-231, HCC1559, BT549, UACC-812, and MDA-MB-453) vs. NBEC	-	MDAMB-231	cell proliferation, G1/S phase transition	miR-214-3p, FOXP2	21
HER2+ breast cancer	Downregulated	Trastuzumab-resistant cells vs. wild-type cells	Mostly distributed in the cytoplasm	Wild-type cells BT-474, Trastuzumab-resistant cells SKBR3	cell viability, apoptosis, colony formation, mammosphere formation, trastuzumab resistance, CSC-like properties, and multiple chemoresistance	miR-100, miR-452, DLG5, PRDM16, MUC4	24
Pancreatic cancer	Upregulated	(AsPC-1, Capan-2, SW1990, PANC-1, PaCa-2, BxPC-3) *vs.* HPDE	-	PaCa-2, PANC-1	cell proliferation, cell invasion	HuR, CTNNB1, WNT/β-catenin signaling pathway	26
Melanoma	Downregulated	BRAF inhibitor-resistant cell lines vs. BRAF inhibitor-sensitive cells (A357P and SKMEL5)	Mainly in the nucleus	A575P, SKMEL5	toxicity response, proliferation, apoptosis	PP1α, MAPK signaling	25
Gastric cancer	Downregulated	(MKN45, MGC-803, AGS, SGC-7901) *vs.* GES-1	Predominately localized in the nucleus	MKN45, MGC803, AGS, SGC-7901	migration, invasion, EMT	hnRNPA1, PKM1, PKM2	23

**Table 2:** Summary of experiments in murine models to study the roles of LINC00589 in tumor development.

Cancer type	Animal models	Groups	Experiment phenotypes	Ref.
Glioma	Four-week-old athymic nude mice (BALB/c)	U87 and U251 cells (control, stably expressing sh-NC+EV, sh-BACH2, sh-FUS, TSLNC8-OE and sh-BACH2+sh-FUS+TSLNC8-OE)	Tumor volume, percent survival analysis	28
HCC	Nude mice	SMCC-7721 cells infected with the lentivirus expressing TSLNC8 or the control	Tumor volume, tumor weight, metastatic nodules in the liver, lung, and intestine	19
Lung cancer	Six-week-old male BALB/c nude (nu/nu) mice	H1975 cells (control, vector, osimertnib + TSLNC8, osimertnib + vector, TSLNC8, osimertnib)	Tumor volume and weight, WB and qPCR of EGFR and phosphorylation of EGFR (Tyr1068) and STAT3 (Tyr705), IHC of EGFR and phosphorylation of STAT3 (Tyr705)	27
HER2+ breast cancer	Female athymic BALB/c nude mice (4-6 weeks)	TR breast cancer cells (Lv-NC, Lv-LINC00589, miR-NC, miR-100 mimic, or miR-452 mimic)	tumor volume, weight, luciferase activity, qPCR and IHC of DLG5 and PRDM16	24
Pancreatic cancer	Nude mice	PaCa-2 cells (Control and TSLNC8-knockdown)	Pulmonary metastatic nodules	26
Melanoma	4-week-old female BALB/c nude mice	TSLNC8-overexpressing or vector-transfected cells into nude mice, and treated daily with 40 mg/kg PLX4720	Tumor weight, tumor volume, WB of p-MEK and p-ERK levels	25
Gastric cancer	Nude mice	MGC803 cells (control shRNA, sh-LINC00589); PMSNs-control and PMSNs-LINC00589 groups	Peritoneal metastatic nodules, IHC of hnRNPA1, PKM2, Ki67, or CD31	23

**Table 3:** Summary of the expression of TSLNC8 and its associations with pathological features and prognosis in clinical samples.

Cancer type	Detection method	Expression (Tumor vs. Normal)	Human tissues	Tumor specimens	Significant clinical variables	End-points (analyze methods)	Prognostic biomarker	Ref.
Glioma	RT-qPCR	Downregulated	80 paired tumor/adjacent noncancerous tissues	80 glioma samples	tumor size, distant metastasis, TNM stage	-	-	20
Glioma	RT-qPCR	Downregulated	12 for each group (normal brain tissues, grade 1-2, 3-4)	-	-	-	-	28
HCC	RNA-seq	Downregulated	TCGA (50 paired tumor/ adjacent noncancerous tissues)	-	-	-	-	19
	RT-qPCR	Downregulated	120 matched tumor/nontumor tissues	120 HCC samples	cancer embolus, tumorous number, differentiation grade	OS (KM plot)	Favorable	
	genomic RT-qPCR	Downregulated	72 paired tumor/nontumor tissues	-	-	-	-	
Lung cancer	RT-qPCR	Downregulated	31 pairs of lung cancer tissues and adjacent normal lung tissues	31 lung cancer samples	gender, lymph node metastasis, TNM stage	-	-	27
Breast cancer	RT-qPCR	Downregulated	10 cancer tissues and normal adjacent tissues	-	-	-	-	21
HER2+ breast cancer	RT-qPCR	Downregulated	Trastuzumab-responding (N= 38) and non-responding (N=33) breast cancer patients	-	-	-	-	24
	ISH	-	-	92 trastuzumab-treated HER2-positive breast cancer samples	TNM stage	OS (KM plot, Multivariate analysis)	Favorable	
Pancreatic cancer	RT-qPCR	Upregulated	70 paired tumor/nontumor tissues	70 pancreatic cancer samples	TNM stage, distant and lymph node metastasis	OS (KM plot)	Unfavorable	26
Melanoma	RNA-Seq	Downregulated	GEPIA-TCGA dataset (tumor=461, normal =558)	-	-	-	-	25
	RT-qPCR	Downregulated	8 paired BRAF inhibitors pretreated and treated samples	-	-	-	-	
Gastric cancer	RT-qPCR	Downregulated	143 paired tumor/adjacent normal tissues	143 gastric cancer samples	N stage, M stage, TNM stage	OS (KM plot, Multivariate analysis)	Favorable	23

## Abbreviations

LncRNAs: Long noncoding RNAs
TSLNC8: Tumor Suppressor LncRNA on Chromosome 8p12
LINC00589: Long Intergenic Non-Protein Coding RNA 589
C8orf75: Chromosome 8 Open Reading Frame 75
ceRNA: Competitive endogenous RNA
EMT: Epithelial-mesenchymal transition
OS: Overall survival
HCC: Hepatocellular carcinoma
TCGA: The Cancer Genome Atlas
DSS: Disease-specific survival
PFI: Progression-free interval
COAD: Colorectal adenocarcinoma
UCEC: Uterine corpus endometrial carcinoma
ACC: Adrenocortical carcinoma
GBM: Glioblastoma multiforme
